# Liposome-Encapsulated Morphine Affords a Prolonged Analgesia While Facilitating Extinction of Reward and Aversive Memories

**DOI:** 10.3389/fphar.2019.01082

**Published:** 2019-09-20

**Authors:** Victoria Gómez-Murcia, Bruno Ribeiro Do Couto, Juan C. Gómez-Fernández, María V. Milanés, María L. Laorden, Pilar Almela

**Affiliations:** ^1^Department of Pharmacology, Faculty of Medicine, University of Murcia, IMIB-Arrixaca, Murcia, Spain; ^2^Department of Human Anatomy and Psychobiology, Faculty of Psychology, University of Murcia, IMIB-Arrixaca, Murcia, Spain; ^3^Department of Biochemistry and Molecular Biology A, Faculty of Veterinary, Regional Campus of International Excellence “Campus Mare Nostrum”, University of Murcia, IMIB-Arrixaca, Murcia, Spain

**Keywords:** liposomes (SUV, LUV), morphine, analgesia, reward memory, aversive memory extinction

## Abstract

Morphine is thoroughly used for pain control; however, it has a high addictive potential. Opioid liposome formulations produce controlled drug release and have been thoroughly tested for pain treatment although their role in addiction is still unknown. This study investigated the effects of free morphine and morphine encapsulated in unilamellar and multilamellar liposomes on antinociception and on the expression and extinction of the positive and negative memories associated with environmental cues. The hot plate test was used to measure central pain. The rewarding effects of morphine were analyzed by the conditioned-place preference (CPP) test, and the aversive aspects of naloxone-precipitated morphine withdrawal were evaluated by the conditioned-place aversion (CPA) paradigm. Our results show that encapsulated morphine yields prolonged antinociceptive effects compared with the free form, and that CPP and CPA expression were similar in the free- or encapsulated-morphine groups. However, we demonstrate, for the first time, that morphine encapsulation reduces the duration of reward and aversive memories, suggesting that this technological process could transform morphine into a potentially less addictive drug. Morphine encapsulation in liposomes could represent a pharmacological approach for enhancing extinction, which might lead to effective clinical treatments in drug addiction with fewer side effects.

## Introduction

Pain is one of the most common symptoms of disease, causing suffering in millions of persons every day. Alleviating pain has always been one of the main objectives of pharmacological research and finding analgesic drugs with reduced secondary effects is of special interest. Morphine, as other opiates, is thoroughly used for pain control in many situations, although it has drawbacks, one of the main ones being possible addiction. Indeed, opioid addiction is one of the most important plagues in modern societies (Nuckols et al., 2014; Cheatle, 2015; Dart et al., 2015).

Despite intensive attempts to develop alternative analgesics, there are currently none to replace opioids in the treatment of severe pain. The requirement for escalating doses to maintain analgesia increases the potential for prescription opioid misuse, diversion, and overdose. New advanced-formulation designs can be used with analgesic drugs to reduce dosing schedules, provide more consistent plasma levels, improve drug absorption, and speed up the therapeutic action beginning. In this sense, opioid liposome formulations, which are known to produce controlled drug release, have been widely tested for decades for pain treatment (Kim et al., 1996). 

It is well known that the quicker a drug accesses the central nervous system, the greater its addictive potential is. Traditionally, this has been thought to be due to the more euphorigenic and/or reinforcing properties, but it is necessary to look into this aspect more thoroughly. Morphine is a substance with a great ability to induce addiction. Liposomes specific pharmacokinetic could affect the addictive process, although very little is known about the effects of morphine encapsulation in this disorder (Samaha and Robinson, 2005).

It has been established that the reinforcing properties of drugs of abuse are partially due to their capacity to increase memory consolidation. New treatments for addiction include the possibility of manipulating learning and memory processes to reduce drug seeking by promoting abstinence and preventing relapse (Everitt, 2014).

The conditioned-place preference (CPP) test has been extensively used to study the rewarding properties of drugs of abuse (Tzschentke, 2007; Garcia-Carmona et al., 2013; Garcia-Carmona et al., 2015). Moreover, the conditioned-place aversion (CPA) test is a highly sensitive animal model for the assessment of the dysphoric and aversive aspects of drug withdrawal, as well as for investigating the neural substrates underlying the negative memory linked to drug withdrawal (Stinus et al., 2000; Myers et al., 2012).

Extinction of this preference/aversion takes place if the association is reduced by repeated exposure to the drug- or withdrawal-related context without the unconditioned stimulus, and the initial response (CPP or CPA) can be Frecovered by a priming dose of drug, stress or by conditioned cues (Tzschentke, 2007). Extinction is finished when animals no longer prefer/avoid the previously cue-paired context. It has been shown that memory reconsolidation requires single context re-exposure, while extinction needs multiple cue re-exposure without the unconditioned stimulus (Power et al., 2006). For example, conditioned fear studies indicate that extinction does not delete the initial context, but the organism knows that the previous stimulus is not caused by this cue (Bouton, 2004). Therefore, extinction requires associative learning, consolidation, and the formation of a new memory (Milad and Quirk, 2002).

With this background, in this experimental work we try to relate the rate of morphine delivery to the brain with changes in memory processes induced by the chronic administration of the drug. Future investigations are needed to establish the specific mechanisms by which differences in the cellular activation process induce differences in the start-up of intracellular signaling cascades, thus promoting neuroplasticity.

## Materials and Methods

### Animals

Adult male Swiss mice weighing 25 to 30 g were housed five to seven/standard cage in an environment with controlled temperature and humidity on a constant 12-h light/dark cycle (8 am–8 pm). Mice were habituated to the experimental room for at least 1 week prior to the experimental starting. Animals received access to water and food *ad libitum*. 

All animal experiments were carried out according with the guidelines provided by the European Communities Council Directive of 24 September 2010 (2010/63/UE) and were approved by the Comité Ético de Experimentación Animal (Universidad de Murcia; RD 53/2013). Protocols were planned to minimize the number of experimental animals and to minimize their suffering. 

### Design of Morphine Liposomes

#### Drugs and Chemical for Liposomes Preparation

Morphine hydrochloride was purchased from Alcaliber Laboratories. Hydrogenated soy phosphatidylcholine (HSPC) and monomethoxy polyethyleneglycol 2000-distearoyl phosphatidyl-ethanolamine (mPEG-DSPE) were a kind gift from Lipoid K.G. (Ludwigshafen, Germany). Cholesterol (CHOL) was purchased from Avanti Polar Lipid, Inc. (Alabama, USA), ethanol from Scharlau (Barcelona, Spain), chloroform from Lab Scan (Sowinskiego, Poland), methanol and ammonium sulfate from Merck KGaA (Darmstadt, Germany), and diafiltration cassette from Thermo Scientific (IL, USA). Dialysis membrane (with MW cutoff of 12,000–14,000 Da), desoxycholate, *tert*-butanol, sodium acetate, and all other chemicals used in this work were purchased from Sigma-Aldrich (Madrid, Spain).

#### Preparation of Pegylated Unilamellar Liposomes

The remote loading technique described by Barenholz and colleagues was used to obtain high entrapment (Gabizon et al., 2003). Lipids were dissolved in chloroform-methanol (2:1) in the molar ratio HSPC:CHOL:mPEG-DSPE (56.4:38.3:5.3). The organic solvent was evaporated under nitrogen current, and the last traces removed by dessication under vacuum for at least 3 h. The dessicated lipids were then dispersed using a vortex after adding a solution of 240 mM ammonium sulfate to form multilamellar vesicles (MLVs). Afterwards, they were extruded by high-pressure through polycarbonate membranes (Whatman Nuclepore, USA). First, they were extruded 10 times through 0.4 μm filters and then 10 more times through 0.1 μm pore size, and finally, liposomes with a narrow particle size distribution were obtained. Following extrusion 1 mL of 40 mg/mL morphine hydrochloride was then added to the liposomal suspension and the mixture was quickly heated for 30 min to 60°C (above of the phase transition) to increase the permeability of the bilayer. The drug external to the liposomes was substituted with 10% sucrose solution through diafiltration cassettes.

#### Preparation of MLVs Using Dehydration–Rehydration Vesicles

Multilamellar vesicles were obtained from HSPC and CHOL in a molar ratio of 2:1, as described (Krugner-Higby et al., 2009). A mixture of 80 μmol of the phospholipid and 40 μmol of CHOL was dried from chloroform solution, dissolved in 1 mL of *tert*-butanol by heating to 60°C in a water bath, frozen in dry ice-isopropanol, and lyophilized for 24 h. The dried lipid was expanded in 1 mL of 240 mM ammonium sulfate for 60 min at 60°C in a water bath. Liposomes were frozen in a dry ice/isopropanol mixture for 2 min, stored at −20°C overnight, defrosted at room temperature, diluted to 5 mL in sterile saline (0.9% NaCl), and placed in centrifuge tubes and sedimented at 10,000 rpm at 4°C for 15 min. The supernatant was removed from the tube and the liposome pellet was resuspended in 1 mL of 40 mg/mL morphine. The suspension was incubated for 1 h in a bath at 60°C, and free morphine was separated by centrifugation at 10,000 rpm for 30 min at 4°C. Finally, the resulting pellet was resuspended in 1 mL of 10 mM sodium acetate buffer, pH 4.0. Immediately prior to injection into animals, pegylated unilamellar liposomes (PEG-L) and MLV were gently stirred and then slowly drawn up into a syringe.

#### Determination of Morphology, Size, and Zeta Potential

Liposomal morphology was visualized by transmission electron microscopy (TEM). TEM was accomplish with a Philips Technai-12 transmission electron microscopy operated at an accelerating voltage of 120 kV, using a camera Megaview II for imaging acquisition. Samples were prepared by dropcasting 10 μL of the formulation solution on a 200-mesh formvar/carbon-coated copper/nickel grid and allowing it to dry at room temperature. Staining was performed using 2% uranyl acetate. Size was measured by laser light scattering 4800 (Zetasizer Nano ZS; Malvern Instruments, Malvern, UK). Zeta potential was measured by using a Malvern Zetasizer Nano ZS (Malvern Instruments) at 25°C.

#### Determination of Liposomes Entrapment Efficiency (EE%) and Drug Loading

Liposomes were dissolved by using 5% desoxycholate. The amount of morphine in each sample was evaluated spectrophotometrically by estimating the absorbance at 285 nm, with a molar absortivity of log e 3.19 (Sangster and Stuart, 1965), and using a standard calibration curve from 0.025 to 0.1 mg/mL. Each experiment was performed in triplicate. EE% was calculated using the following equation: 

$$\text{\% Entrapment efficiency}=\frac{\text{Amount of morphine entrapped with in liposomes}}{\text{Total amount of morphine used for synthesis}}\;\times \text{100}$$

Drug loading was calculated according to:

$$\text{\% Drug loading }=\frac{\text{Amount of morphine in liposomes}}{\text{Amount of morphine in liposomes}+\text{ Amount of lipid used }}\times \text{ 100}$$

#### *In Vitro* Release Studies

*In vitro* studies were carried to compare PEG-L and MLV. *In vitro *studies were performed in pH 7.4 phosphate buffer at 37°C. Stability studies were performed with the same solutions used in liposomes (sodium acetate for MLV and 10% of sucrose for PEG-L) at 4°C. T*he assay at this temperature was done in order to evaluate the stability of these liposomes during storage*. 1.5 mL of suspension was placed inside the dialysis bag, tied at both ends, and dipped in the dissolution medium for both studies. Suspensions were then placed on an electronic shaker set at 100 rpm. At different time points, 1 mL of release medium was eliminated and replaced with the same volume of fresh medium. Analysis was carried out using a UV spectrophotometer set at 285 nm.

#### Continuation of *In Vitro* Drug Release Studies

Ten microliters of internal standard (d3-morphine) and 4 mL of 100 mM phosphate buffer (pH 6) were added to 1 mL of plasma. C8 cationic exchange columns Bond Elute Certify (Agilent Technologies) were activated with 2 mL of methanol and then conditioned with 2 mL of 100 mM phosphate buffer (pH 8–9). Next, samples were applied at a 1 to 2 mL/min flow rate to the SPE cartridge; all elution solvent was forced out to pass. Then, columns were rinsed sequentially with 2 mL of bidistilled water, 2 mL of 100 mM acetate buffer (pH 4) and 2 mL of methanol. Elution was developed with 2 mL of methanol/ammonium hydroxide (98:2 v/v). The eluate was evaporated to dryness using a dry bath, heating at 38°C; 50 μL of MSTFA (*N*-methyl-*N*-trimethysilil-trifluoroacetamide) was added as a derivatization reagent; and the mixture finally heated to react at 70°C for 20 min. Two microliters of residue was injected onto the gas chromatography system (Agilent Technologies 7890B).

### *In Vivo* Drug Release Studies

Mice (n = 4–6 per group) received one intraperitoneal (i.p.) administration of 60 and 90 mg/kg morphine liposomes, either PEG-L or MLVs or free morphine. Animals were anesthetized with pentobarbital (100 mg/kg, i.p.) and blood samples (cardiac puncture and terminal blood sampling) were drawn at different times (0.0833, 0.5, 2, 4, 6, 8, and 24 h) after drug administration. Heparinized blood samples were centrifuged, and plasma was fresh-frozen and stocked up immediately at −80°C until use. Plasma samples were purified by a liquid-solid extraction method (Krugner-Higby et al., 2009) and injected onto a gas chromatography system (Agilent Technologies 7890B) equipped with a mass-selective detector (Agilent Technologies 5977A MSD) and an autoinjector Gerstel MPS 2XL. 429 and 432 ions (specific for morphine and d3-morphine, respectively) were selected for quantification. The calibration curve was linear over a concentration range from 5 to 2,000 ng/mL plasma.

Plasma concentration-time data were pharmacokinetically analyzed for each sample via a PKSolver program (Zhang et al., 2010). The model used to analyze pharmacokinetic parameters was the “noncompartmental model after an extravasal input.” This methodology requires less assumptions than model-based approaches and is readily automated, and minimal intervention and decision making are required from the user (Gillespie, 1991; Gabrielsson and Weiner, 2012).

### Antinociception Measurement of the Liposomal Formulations

Experiments were performed after an i.p. injection of free morphine or the liposomal formulations by subjecting animals to a standard hot plate testing (Socrel DS37, Ugo Basile, Italy) using a platform (25 × 25 cm) which was maintained at 52 ± 0.2°C, and a transparent cylinder (19 cm diameter × 18 cm height) used to prevent mice from escaping. Animals stayed on the platform until they licked their forepaws or the time established as cutoff (60 s). A baseline was obtained for each animal prior to any drug administration. Animals were neither handled nor habituated to the experimental place.

After completing the baseline testing (same day of the test), any antinociceptive effects were measured at specific time points by subjecting the animals to the hot place testing again. For this purpose, animals were separated into different groups depending on the morphine dose and treatment conditions: 60 and 90 mg/kg for each liposome formulation and free morphine. Antinociception was determined in six to eight animals at each dose and for each group at different times after drug administration.

Latency (the time taken to respond to the heat stimulus) was evaluated as the time taken by the animal to lick one of its rear paws or until the cutoff time was reached. Antinociception was determined by the percentage of maximal potent effect (MPE), which was calculated as: 

$$\text{\%MPE}=\frac{\text{Postdrug latency}-\text{baseline}}{\text{cut}-\text{off time}-\text{baseline}}\times \text{ 100}$$

### Conditioned-Place Preference Protocol

The rewarding properties of morphine were measured using the CPP paradigm. Mice were conditioned and tested during the light cycle phase in the CPP apparatus as described previously (Garcia-Carmona et al., 2015). The CPP equipment used in this study consisted of two identical boxes with three polyvinyl carbonate chambers (Valverde et al., 1996) connected to a computer. Two side chambers (length, 20 cm; width, 18 cm; height, 25 cm; spaced at 4 cm from each other) were separated by a smaller chamber (length, 20 cm; width, 7 cm; height, 25 cm). The two larger chambers differed in their wall paint and floor texture and provided distinct contexts. Manual guillotine doors were inserted during the conditioning sessions and removed during the tests.

The CPP procedure consisted of three phases: preconditioning test, conditioning phase, and postconditioning test. 

*Preconditioning test (day 0):* During this phase, each mouse freely explored the CPP apparatus for 15 min (n = 32) and the time spent in each compartment was recorded by a computer program (CPP Win 2.0. Panlab, Barcelona, Spain). After the session, animals were randomized to be assigned to free drug, drug encapsulated into liposomes, or saline administration groups and to be assigned to a particular compartment. Animals spending less than 6 min in either the white or black compartment were considered not to be neutral in preference for either side, and were eliminated from further studies (n = 3).

*Conditioning phase (days 1–6):* Mice were treated for eight consecutive days. To evaluate the rewarding effects of free and encapsulated morphine, the mice received an injection of morphine hydrochloride (60 mg/kg, i.p.), nonencapsulated (free-morphine group) or encapsulated into liposomal formulations (MLV or PEG-L) on days 1, 3, and 5, and an injection of saline solution on days 2, 4, and 6. Like that, mice were assigned to one compartment on odd days and to the opposite compartment for even days. The control group received saline every day. Immediately after morphine or saline administrations, the animals were put in the assigned compartment, and then doors matching the walls of the compartment were closed for 1 h. This compartment will become associated with the motivational effects of the drug.

*Postconditioning test (day 7)*: This test was conducted in the same way as the preconditioning phase (free access to both compartments for 15 min), 24 h after the final conditioning session.

The CPP score represents the time spent by mice in the drug-paired chamber during the testing phase minus that spent during the preconditioning phase.

*CPP Extinction Test:* A study of the extinction of morphine place preference was also performed. This consisted of allowing mice daily access to both compartments for 15 min until no significant differences were observed between time spent in the preconditioning and postconditioning test on two consecutive days. The score represented each day consists of a mean of the score for each animal. 

The weight gain of mice was controlled during the treatment since chronic morphine treatment is known to induce a reduction in body weight gain due to lower caloric intake (Houshyar et al., 2004).

### Conditioned-Place Aversion Protocol

Negative affective symptoms associated with morphine withdrawal were examined by the CPA paradigm, a behavioral test frequently used to analyze the affective-like responses of drug withdrawal in rodents. The place conditioning apparatus is described above. The CPA schedule consisted of three phases: preconditioning test, conditioning phase, and postconditioning test. 

*Preconditioning Test (Day 0):* See CPP Section. Thirty-Two Animals Were Used, and Three Were Excluded From Further Studies.

*Conditioning phase (days 1–4):* Mice were randomly assigned to receive saline (control group), free morphine, PEG-L, or MLV. Morphine was injected every 12 h (at 8 am and 8 pm) with a chronic escalating dose regimen according to the following protocol: day 1, 10 mg/kg; day 2, 30 mg/kg; day 3, 50 mg/kg; day 4, 60 mg/kg (only one injection in the morning). The control group received saline instead of morphine. On day 4, and 1 h after the last morphine injection, one compartment of the place aversion apparatus was randomly chosen to be paired to naloxone, subcutaneously (s.c.) administered at a dose of 1 mg/kg (morphine-treated group and saline-treated group). Immediately after naloxone injection, animals were placed in the assigned compartment, and then the doors matching the walls of the compartment were closed for 18 min. The environment will come to elicit avoidance-withdrawal due to negative state associated with naloxone.

*Postconditioning Test (Day 5):* See CPP Section.The CPA score depicts the time spent in the naloxone-paired compartment during the testing phase minus that spent during the preconditioning phase.

*Physical Syndrome of Morphine Withdrawal:* On day 4 of the CPA schedule, animals were weighted before and 18 min after naloxone injection. Their behavior was video-recorded for 18 min using white light. The experimenter, blinded with respect to animal treatments, analyzed the videotapes to assess behavioral patterns. A score was calculated using a modification of the scale described by Gellert and Holtzman (Gellert and Holtzman, 1978), in which two classes of signs were differentiated. Graded signs were considered as weight loss (each 1% of weight loss quantified as 1); number of jumps (1–4 quantified as 1, 5–9 quantified as 2, and >10 quantified as 3); and number of body shakes (1–2 quantified as 2, >3 quantified as 4). Checked signs, in which only the presence or absence was evaluated, were diarrhea (Cheatle, 2015) and ptosis (Cheatle, 2015). The behaviors were assessed based on the description of Fernández-Espejo et al. (Fernández-Espejo et al., 1995).

*CPA Extinction Test:* A study of the extinction of morphine place aversion was also performed as described in CPP section. 

### Statistical Analysis

Data are represented as mean ± standard error (SEM) and were analyzed using one-way analysis of variance (ANOVA). The Newman-Keuls *post hoc* test was used to identify individual mean differences. Student’s two-tailed *t* test was used when comparisons were limited only to two experimental groups. Differences with a *P* < 0.05 were considered significant. Statistical analysis that involved performing a two-way ANOVA with repeated measures followed by post hoc analysis (Tukey’s test) was used when multiple measures over time within three experimental groups were compared. Statistics were performed using the GraphPad Prism 6 statistical package (GraphPad Software Inc., San Diego, CA, USA). 

## Results

### Liposomes Formulations Ensure an Extended-Release Drug Delivery

**Figure 1:**
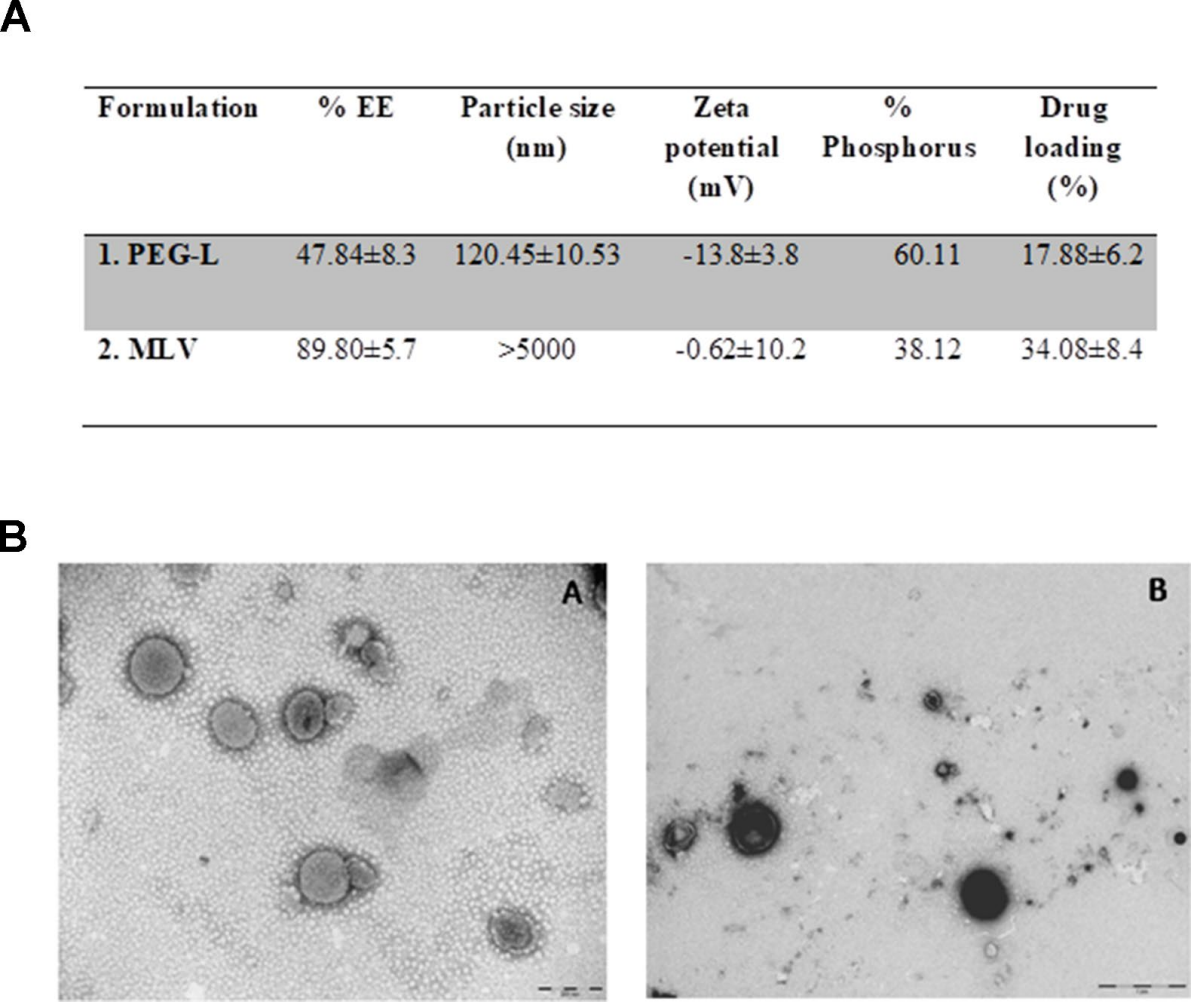
**(A)** Characterization of the liposomes. EE stands for encapsulation efficiency. **(B)** Transmission electron microscopy (TEM) pictures of the liposomes used in this work. Left panel shows PEG-L **(A)**, whereas right panel shows MLV **(B)**. Scale bar represents 200 nm **(A)** and 1 µm **(B)**.

Characterization of liposomes regarding entrapment efficiency, size, morphology, and zeta potential is shown in **Figure 1**. **Figure 1A** shows that a higher percentage of morphine entrapment (89.80 ± 5.7%) and higher drug loading (34.08 ± 8.4%) was achieved in MLV due to its higher size (>5,000 nm). The vesicle size distribution for PEG-L tested was unimodal with an intensity-average size of 120.45 ± 10.53 nm, whereas MLV showed a bimodal curve with higher heterogeneity. Morphine entrapment in the case of PEG-L was 47.84 ± 8.3, with a drug loading of 17.88 ± 6.2%, lower than with MLV. The zeta potential was also better in PEG-L with −13.8 ± 3.8 mV, whereas MLV presented a zeta potential next to zero (−0.62 ± 10.2).

**Figure 1B** shows TEM images of both types of liposomes loaded with morphine. The left panel of this figure (**Figure 1BA**) shows an image of PEG, while the right panel (**Figure 1BB**) shows an image of MLV. Both of them have a closed and spherical morphology. The vesicles size, obtained by TEM, usually correlates well with the average diameter achieved by the laser scattering method. While **Figure 1BA** shows a good homogeneity with respect to the size distribution of PEG-L, **Figure 1BB** image corresponding to MLV shows a large distribution (a non mono-disperse) of liposomes.

The zeta potential is used as an indicator of the stability of colloidal dispersions as it is the case of liposome suspensions. A negative or positive zeta potential indicates that liposomes do not have a tendency to floculate, and they will be able to play their role as vehicles of drugs *in vivo*. On the other hand, the size of liposomes and their uniformity are also indicators of their stability and of their lack of tendency to aggregate.

**Figure 2:**
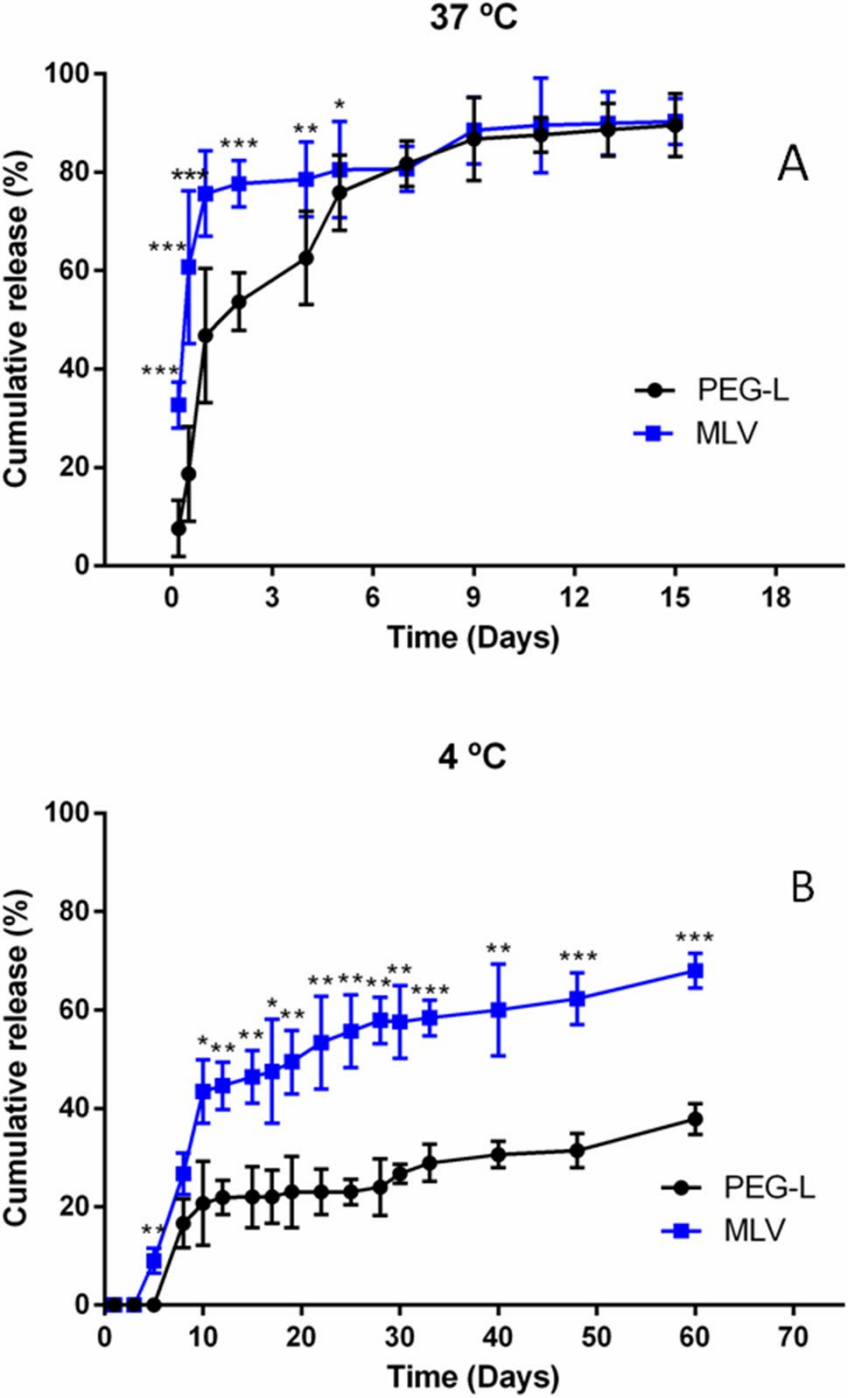
**(A)** Cumulative morphine release from unilamellar (PEG) and multilamellar (MLV) liposomes at 37°C in PBS. Results shown are the mean ± SEM from at least five batches of liposomes. **(B)** Cumulative morphine release from unilamellar (PEG) and multilamellar (MLV) liposomes at 4°C in buffer. Results shown are the mean ± SEM from at least five batches of liposomes.

The release of morphine from liposomes was also studied *in vitro*, to check their stability. **Figure 2A** presents the drug release profiles obtained at 37°C by the dialysis method. Polymer modification of lipids could influence the stability of resulting liposomes, thus, MLV showed a notable initially faster pattern of about 76% liberation during the first day whereas the release from PEG-L during this first day was of only 43%, followed by a sustained release of morphine of both types of liposome samples with no statistically significant differences between them after the 6th day.

The long-term stability of both formulations was investigated upon storage at 4°C for 60 days. As illustrated in **Figure 2B**, formulations showed an initial fast release of drug in both formulations for the first 10 days, amounting to 45% in the case of MLV and to only 18% for PEG-L. After the first 10 days, the rate of release was similar for both preparations, and after 60 days, the cumulative release was 62% for MLV and 33% for PEG-L.

A comparative pharmacokinetic study between free morphine, MLV (multilamellar liposome vesicles) and PEG-L (polyethylene glycol modified unilamellar liposomes) was performed by determining morphine concentration in mice plasma after i.p. administration of 60 or 90 mg/kg (**Table 1**). The AUC (area under the curve) versus time was 20,871.89 ± 162.78, 15,278.47 ± 205.11, and 1,987.38 ± 110.83 ng/h*mL for PEG-L, MLV, and free morphine, respectively, at 60 mg/kg (*P* < 0.001 free morphine versus PEG-L and MLV, and *P* < 0.01 versus MLV). In the present study, PEG-L resulted in a higher Cmax (the maximum plasma concentration that a drug achieves in a specific compartment) of 1,438 ± 6.32 ng/mL, with sustained plasma concentration of morphine, whereas MLV presented a similar Cmax (1,533 ± 17.89 ng/mL) with a faster release than PEG-L. Free morphine showed the lowest t1/2 (0.86 ± 0.43 h) *P* < 0.001 versus PEG-L (7.06 ± 0.91 h) and MLV (7.40 ± 2.26 h). Finally, free morphine presented a Cmax of 1,645 ± 11.82 ng/mL with no significant changes compared with PEG-L and MLV. The blood area under the first moment curve (AUMC) 0-∞ of PEG-L was much higher than for MLV; and even though 1.44-fold greater and 87.11-fold greater than free morphine, indicating that PEG-L improves bioavailability compared with MLV and particularly free morphine (*P* < 0.001 versus PEG-L and MLV, and *P* < 0.001 versus MLV).

**Table 1:** Pharmacokinetic parameters. Plasma concentrations of morphine MLV and PEG-L after intraperitoneal administration of 90 mg/kg in mice are shown. Values are expressed as mean ± SD, parameter derived from the kinetic profile using a compartmental approach. n = 4–6. Data are the mean ± SEM.

Pharmacokinetic parameters	60 mg /kg			90 mg/kg		
	**PEG-L**	**MLV**	**Free morphine**	**PEG-L**	**MLV**	**Free morphine**
**C_max_ (ng/mL)**	1,438 ± 6.32	1,533 ± 17.89	1,645 ± 11.82	2,253 ± 16.56	2,012 ± 21.62	1,927 ± 11.75
**t_½_ (h)**	7.06 ± 0.91***	7.40 ± 2.26***	0.86 ± 0.43	8.30 ± 1.07**, +	11.23 ± 1.75***	0.83 ± 0.78
AUC_0-∞_ (ng/h*mL)	20,871.89 ± 162.78***, ++	15,278.47 ± 205.11***	1,987.38 ± 110.83	39,052.72 ± 456.01***, ++	29,500.91 ± 59.50***	5,358.54 ± 408.42
AUMC_0-∞_ (ng/mL*h ^2^)	204,833.36 ± 843.82***, +++	146,196.83 ± 1,795.92***	2,351.27 ± 62.94	473,653.57 ± 664.51***	430,987.05 ± 1,081.31***	8,357.86 ± 1,607.63
**K_e(1/2)_ (1/h)**	0.09 ± 0.01***	0.09 ± 0.01***	0.81 ± 0.075	0.08 ± 0.00***	0.06 ± 0.00***	0.83 ± 0.05

**P < 0.01, ***P < 0.001 vs free morphine. +P < 0.05, ++P < 0.01, +++P < 0.001 vs MLV. Area under the curve (AUC), area under the first moment curve (AUMC).

In the case of 90 mg/kg, the highest Cmax was for PEG-L (2,253 ± 16.56 ng/mL) followed by MLV and free morphine (2,012 ± 21.62 and 1,927 ± 11.75 ng/mL, respectively) although with no significant differences were detected between them. At this dose, the AUMC 0-∞ was substantially higher for MLV and PEG-L (430,987.05 ± 1,081.31 and 473,653.57 ± 664.51 ng/mL*h^2, respectively) than for free morphine (8,357.86 ± 1,607.63 ng/mL*h^2) (*P* < 0.001 versus PEG-L and MLV). Free morphine exhibited a t1/2 (0.86 2.83 ± 0.78 h) significant lower (*P* < 0.001) versus PEG-L (8.30 ± 1.07 h) and MLV (11.23 ± 1.75 h), but with significant differences between MLV and PEG-L (*P* < 0.05).

**Figure 3:**
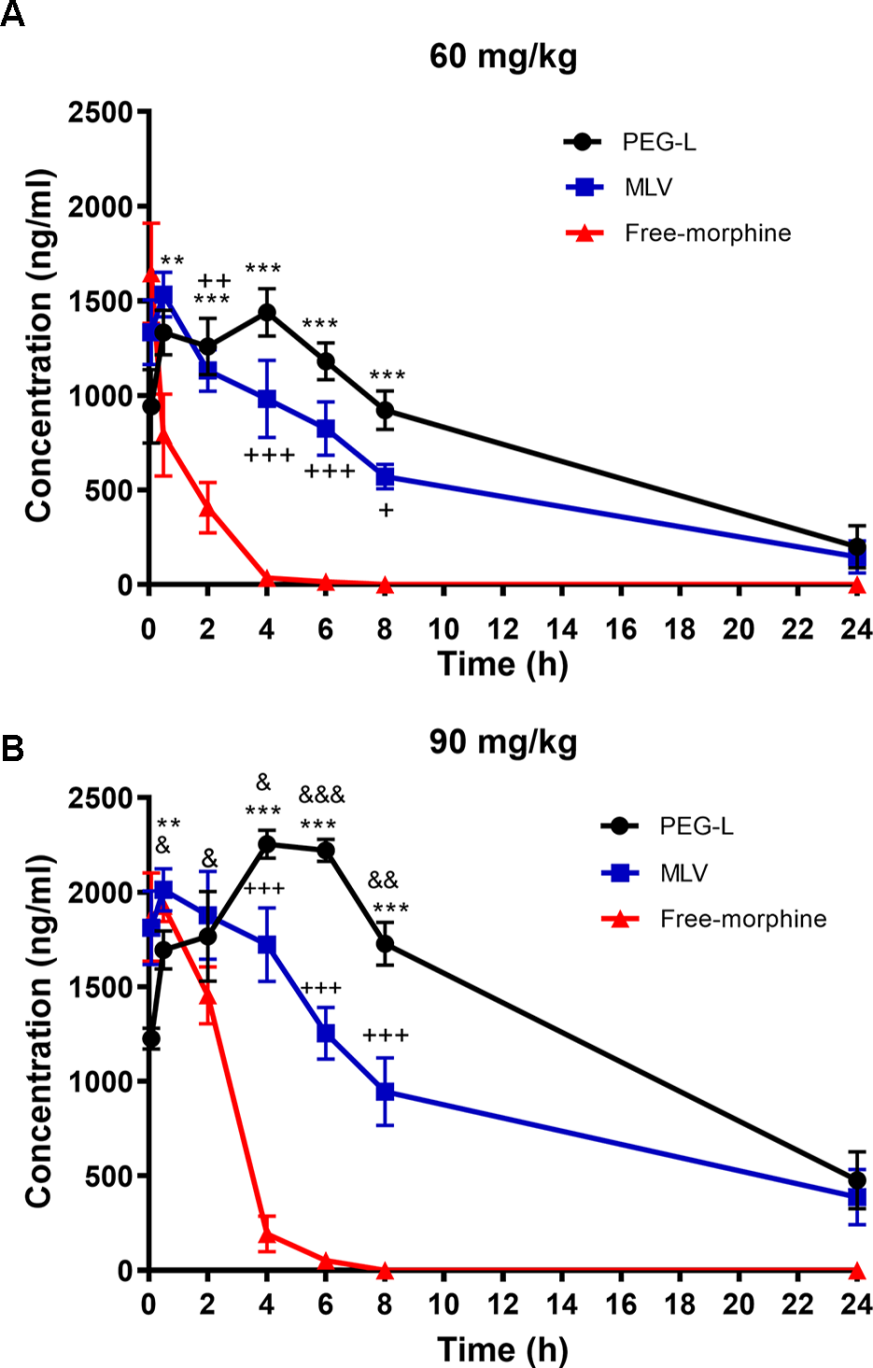
Liposomes formulations ensure an extended-release drug delivery. **(A)** Morphine plasma pharmacokinetics in mice (n = 4–6) receiving i.p. administration of 60 or **(B)** 90 mg/kg of free morphine, MLV or PEG-L. Data are the mean ± SEM. **P < 0.01, ***P < 0.001 versus free morphine; ^+^*P* < 0.05, ^++^ *P* < 0.01, ^+++^ *P* < 0.01 versus free morphine; ^&^ *P* < 0.05, ^&&^ *P* < 0.01, ^&&&^ *P* < 0.001 versus MLV.

Based on previous studies on antinociception (Grant et al., 1995) (Grant et al., 1994) using liposomes to encapsulate high doses of morphine (doses interval 130–1650 mg/kg), much more reduced doses were chosen trying to reflect what would be expected in a pharmacological use, those doses are in the range of doses used in the behavioral studies. Other authors like Hassanipour et al. (Grant et al., 1994) used doses in the range of 50 to 75 mg/kg. Mansouri et al. (Sverrisdottir et al., 2015) used a dose of 50 mg/kg. Varela et al. (Maherani et al., 2011) used doses up to 50 mg/kg. Lamberts et al. (Hernandez-Caselles et al., 1990) used a dose of 128 mg/kg. Many other examples are available in the literature, showing the variety of doses used for this kind of experiments, doses that differ considerably from human posology. **Figure 3** shows the high rapid delivery of free morphine at both doses, while, in the case of MLV and PEG-L, morphine still remained in circulating blood 24 h after injection.

Regarding the pharmacokinetic study at 60 mg/kg, two-way ANOVA revealed a main effect of kind of formulation [*F*(2,36) = 34.23, *P* < 0.0001] and time [(*F*(5,36) = 16.65, *P* < 0.0001)] with a significant interaction between kind of formulation and time [*F*(10,36) = 7.446, *P* < 0.0001)]. As shown in **Figure 3A**, between 2 and 8 h, statistically significant differences (*P* < 0.05, *P* < 0.01, and *P* < 0.001) were found between PEG-L or MLV and free morphine and from 6 to 8 h differences were found between PEG-L and MLV (*P* < 0.05).

Two-way ANOVA at 90 mg/kg showed a main effect of kind of formulation [*F*(2,38) = 60.37, *P* < 0.0001] and time [(*F*(5,38) = 17.53, *P* < 0.0001)] with a significant interaction between kind of formulation and time [*F*(10,38) = 18.05, *P* < 0.0001)]. As shown in **Figure 3B**, significant differences were found from 4 to 8 h when comparing PEG-L vs free morphine (*P* < 0.001), MLV vs free morphine (*P* < 0.01 and *P* < 0.001) and PEG-L vs MLV (*P* < 0.05, *P* < 0.01 and *P* < 0.001).

### Liposomes Formulations Produce a Longer-Lasting Analgesic Effect Compared to the Free Drug

Control analgesia experiments with empty liposomes formulation of PEG-L and MLV were tested with no antinociceptive effect (**Supplementary Material panels A and B**). Moreover, to demonstrate that analgesia induced by liposome-encapsulated morphine can be reversed by an opiate antagonist, after obtaining a baseline for each animal, mice received a s.c. dose of 5 mg/kg naloxone and divided in two groups. 20 min after the antagonist injection, each group received a single dose of 60 mg/kg morphine encapsulated in PEG-L or MLV. None of the mice presented any antinociceptive effect compared to liposome-encapsulated morphine (**Supplementary Material panels C and D**).

**Figure 4:**
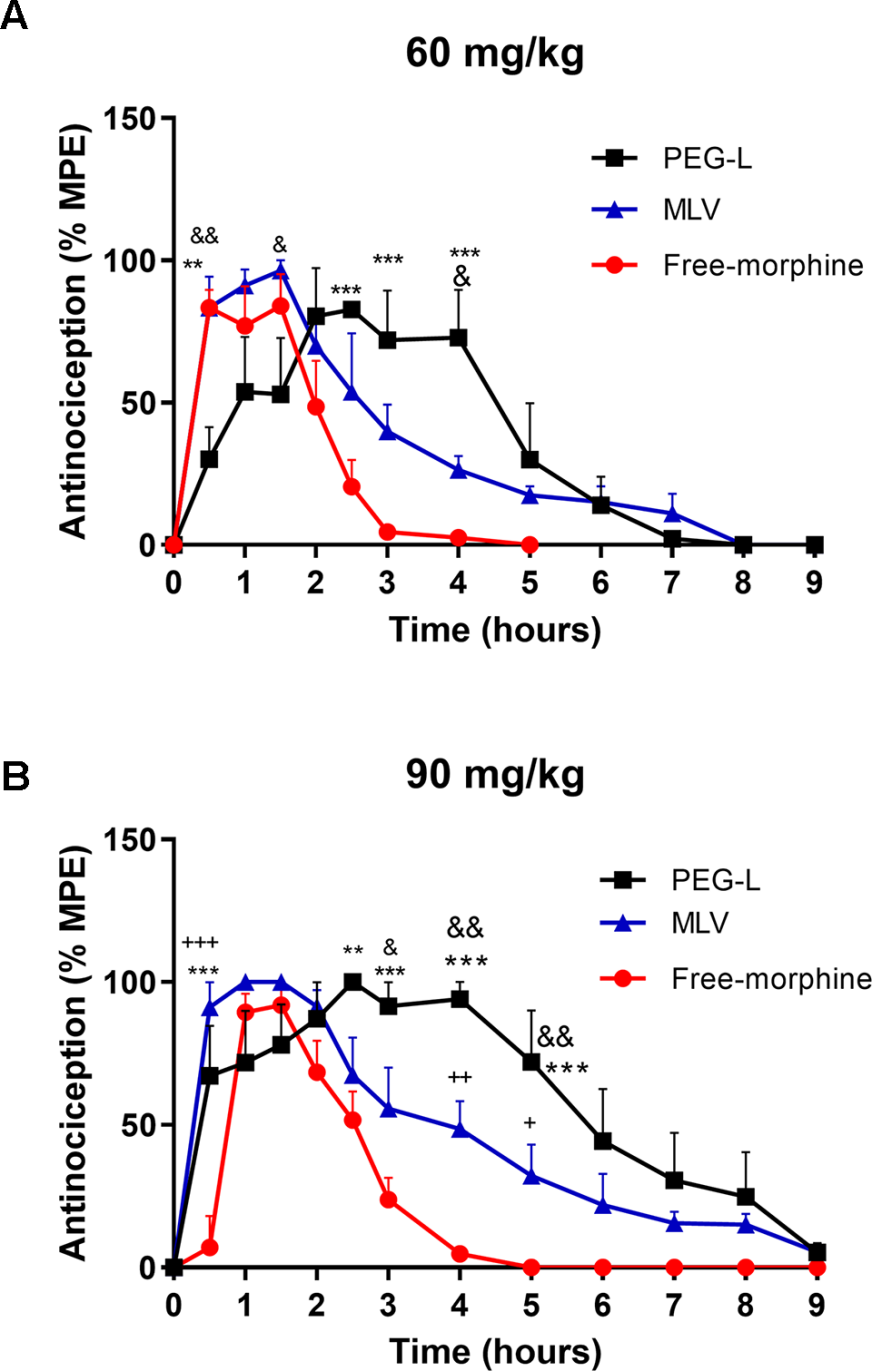
Liposomes formulations produce a long-lasting analgesic effect compared to the free drug. **(A)** Antinociceptive effect as a function of time after a single i.p. administration of free morphine, PEG-L or MLV-L at 60 and **(B)** 90 mg/kg doses. The intensity of analgesia is expressed as % MPE (maximal potent effect). Each data point represents the average for five to eight animals. Data are expressed as mean ± SEM. **P < 0.01, ***P < 0.001 versus free morphine; ^&^ *P* < 0.05, ^&&^ *P* < 0.01 versus MLV; ^+^ *P* < 0.05, ^++^ *P* < 0.01, ^+++^ *P* < 0.001 versus free morphine.

The analgesia produced after the i.p. administration of PEG-L, MLV, and free morphine (60 and 90 mg/kg) is shown in **Figures 4A, B**. Free morphine at 60 mg/kg analgesia peak was reached at about 30 min, and it started to decrease at about 1.5 h along with MLV. However, for free morphine, the decrease of the curve was faster than for MLV, which continued to slowly but steadily decreased.

For PEG-L, there was a significant rightward shift and a maximum of analgesia at 2.5 h, approximately, when MLV and free morphine started to fall, and the analgesic curve gradually decreased over the time. At 60 mg/kg, both liposome formulations increased the duration of analgesia, reaching 8 h, while the analgesic effect was prolonged up to 5 h in the case of free morphine (**Figure 4A**).

On the other hand, when 90 mg/kg was used, PEG-L and MLV analgesia was maintained longer and fell steadily until approximately 9 h compared with free morphine (4 h) (**Figure 4B**).

Regarding the analysis of analgesia at 60 mg/kg, two-way ANOVA revealed a main effect of kind of formulation [*F*(2,139) = 7.469, *P* = 0.0008] and time [*F*(9,139) = 18.24, *P* < 0.0001) with a significant interaction between kind of formulation and time [*F*(18,139) = 4.080, *P* < 0.0001). As shown in **Figure 4A**, statistically significant differences (*P* < 0.01 and *P* < 0.001) were found between PEG-L and free morphine and between PEG-L and MLV (*P* < 0.05,* P* < 0.01).

Two-way ANOVA at 90 mg/kg showed a main effect of kind of formulation [*F*(2,244) = 41.58, *P* < 0.0001] and time [(*F*(11,244) = 30.42, *P* < 0.0001)] with a significant interaction between kind of formulation and time [*F*(22,244) = 4.101, *P* < 0.0001)]. As shown in **Figure 4B**, significant differences (*P* < 0.05, *P* < 0.01 and *P* < 0.001) were found between PEG-L or MLV and free morphine and between PEG-L and MLV (*P* < 0.05,* P* < 0.01).

### Morphine Encapsulation in Liposomes Reduces CPP Duration but Not CPP Expression

**Figure 5:**
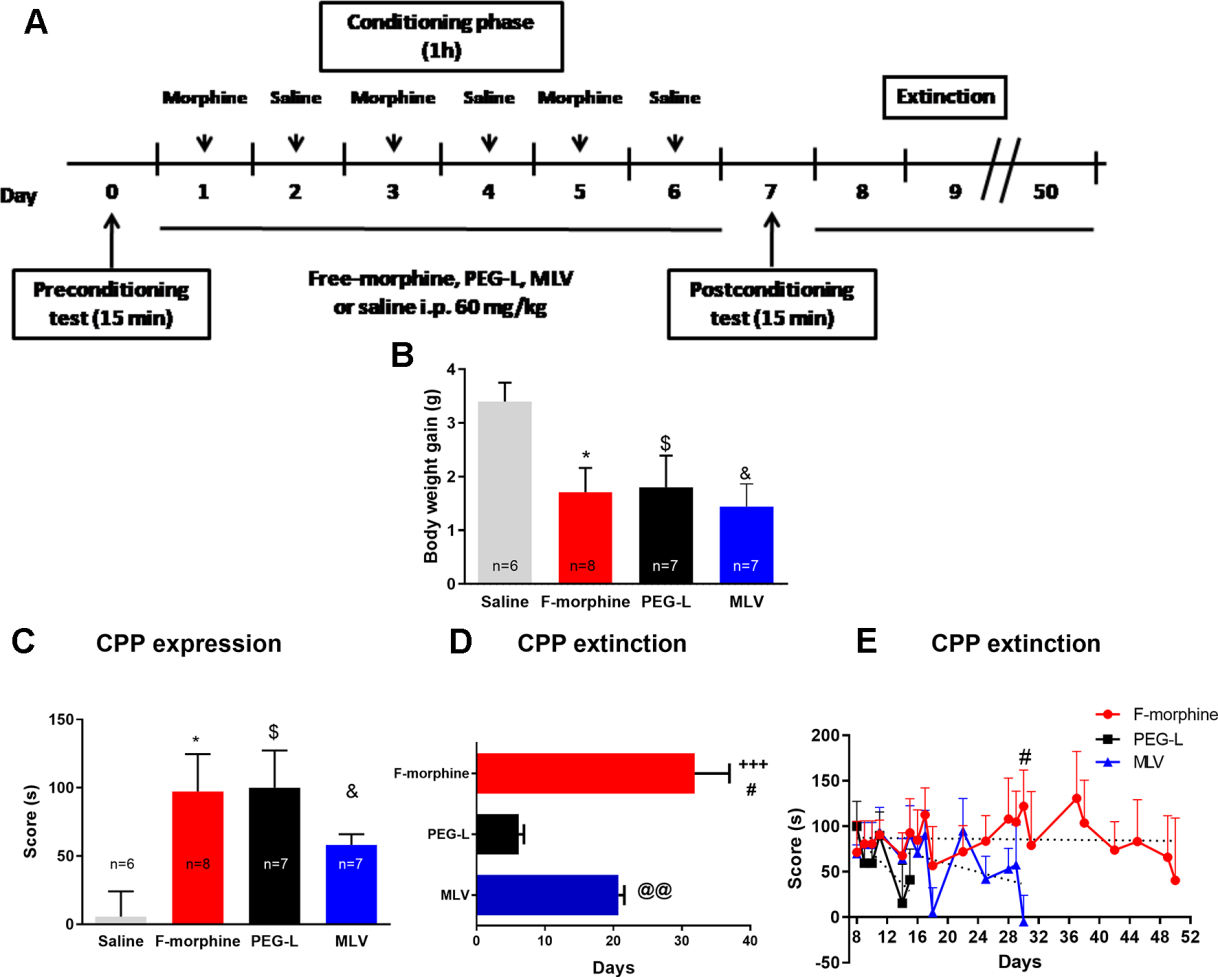
Morphine encapsulation in liposomes reduces the duration of conditioned-place preference (CPP) but not of CPP expression. **(A)** Experimental schedule for drug treatment for CPP test. Mice were treated with chronic morphine/saline for 6 days. Drug was administered encapsulated into MLV or PEG-L or in its free form (60 mg/kg; n = 8 per group). **(B)** Effect of morphine, MLV, or PEG on body weight gain. **(C)** Effect of repeated saline or morphine injection on CPP. The score was calculated for each mouse as the difference between postconditioning and preconditioning time spent in the drug-paired compartment. **(D)** Maintenance of the CPP induced by 60 mg/kg of morphine, MLV, or PEG in mice. The bars represent the number of days that each mouse needed to achieve a complete extinction of the CPP (defined as a lack of significant differences with respect to preconditioning values). Data are the mean ± SEM. ^+++^ *P* < 0.001 versus PEG-L; ^#^ *P* < 0.05 versus MLV; ^@@^ *P* < 0.01 versus PEG-L. **(E)** Extinction of CPP training. Preference scores from day 8 to 50 are shown. Data are the mean ± SEM. ^*, $, &^ *P* < 0.05 versus saline; ^#^ *P* < 0.05 versus MLV.

In the CPP experiments (**Figure 5A**), the weight of the animals was recorded from the pretest day (day 0) until the postconditioning day (day 7). Animals treated with free morphine, PEG-L, and MLV showed a significant [*F*(3,25) = 3.331; *P* < 0.05] lower body weight gain than saline-treated animals (**Figure 5B**), which may be due to a reduced food intake during morphine treatment. Moreover, no significant body weight gain between free morphine, PEG-L, and MLV groups was observed.

Morphine administration, either in its free form or encapsulated in MLV and PEG-L, induced a significant [*F*(3,23) = 3.431; *P* < 0.05] place preference for the drug-paired context in comparison with the saline group (**Figure 5C**). Besides, one-way ANOVA for the CPP score showed no differences between free morphine, MLV, and PEG-L.

To evaluate the persistence of CPP in free morphine–, PEG-L– and MLV-treated animals, the test was assessed every day until no significant differences in the time spent in the morphine-paired room during two consecutive days, compared to the pretest day, were observed. Animals receiving free morphine presented a preference for the drug-paired compartment for a longer time (31.88 days), followed by MLV (20.75 days) and PEG-L (6.14 days) (**Figure 5D**).

One-way ANOVA revealed a significant increase in extinction days [*F*(2,20) = 16.45; *P* < 0.001] with significant differences of free morphine (*P* < 0.001, and *P* < 0.05) comparing to PEG-L and MLV, and a significant difference (*P* < 0.01) between MLV and PEG-L (**Figure 5D**). These results also indicate that the necessary time to extinguish CPP was 1.87-fold higher in the free-morphine group, compared to MLV, and 5.38-fold higher than PEG-L. When comparing both liposome formulations, MLV produced longer preference (2.88 fold) than PEG-L. Two-way ANOVA showed a main effect of kind of formulation [*F*(2,300) = 18.42, *P* < 0.001] with no significant effect of day [*F*(14,300) = 1.5, *P* = 0.1095] or interaction between kind of formulation and day [*F*(28,300) = 0.9916, *P* = 0.4812]. Post hoc analysis showed a significant increase in the preference retained, on day 30 of free morphine–treated animals compared to MLV-treated animals (*P* < 0.05) (**Figure 5E**).

### PEG-L–Treated Animals Manifest Fewer Signs of Physical Dependence

**Figure 6:**
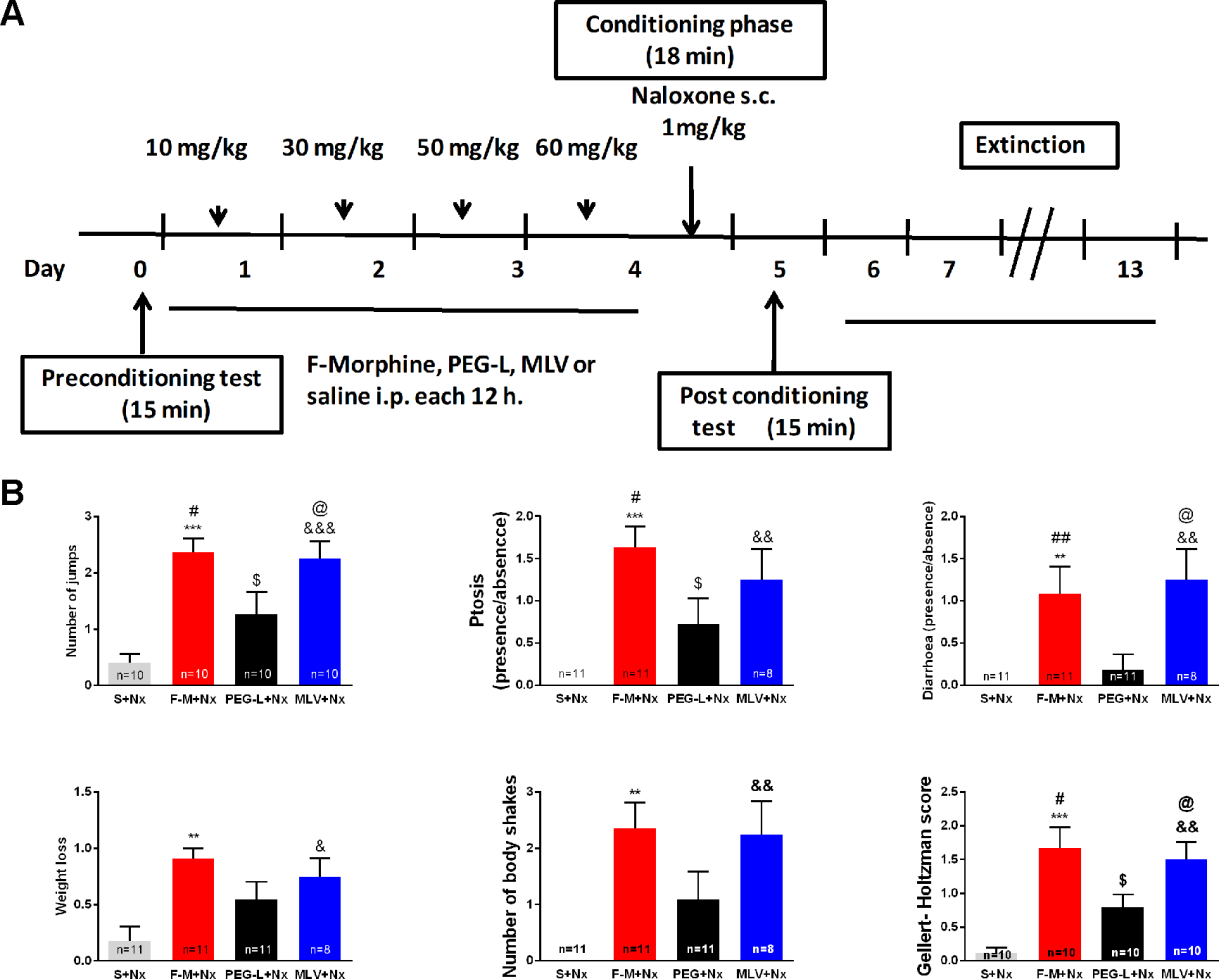
PEG-L–treated animals manifest fewer signs of physical dependence. **(A)** Experimental schedule for drug treatment for conditioned-place aversion (CPA) test. Mice were treated with chronic morphine/saline for 4 days and received an acute injection of naloxone (1 mg/kg, s.c.). Morphine was administered encapsulated into MLV or PEG-L or in its free form (10–60 mg/kg). **(B)** Appearance of signs of morphine withdrawal and Gellert-Holtzman score in animals treated with morphine for 15 min after the conditioning sessions with naloxone. Data are the mean ± SEM. **P < 0.01, ***P < 0.001, ^$^ *P* < 0.05, ^&^ *P* < 0.05, ^&&^ *P* < 0.01, ^&&&^ *P* < 0.001 versus S+Nx; ^#^ *P* < 0.05, ^##^ *P* < 0.01, ^@^ *P* < 0.05 versus PEG-L+Nx. Saline (S), naloxone (Nx), free morphine (F-M); n = 10–11).

In the study of the physical signs of morphine withdrawal (**Figures 6A, B**), one-way ANOVA showed a significant (*P* < 0.01 and *P* < 0.001) increase in weight loss [*F*(3,37) = 5.788], number of jumps [*F*(3,36) = 9.902], number of body shakes [*F*(3,37) = 6.896], ptosis [*F*(3,37) = 8.228], diarrhea [*F*(3,37) = 6.661] and Gellert-Holtman score [*F*(3,16) = 10.26] in animals receiving free morphine and naloxone, compared to the control animals (saline + naloxone). A similar increase (*P* < 0.05, *P* < 0.01, and *P* < 0.001) was observed in all these parameters in the MLV group compared with control animals. However, PEG-L–treated animals only showed a significant (*P* < 0.05) increase in the number of jumps, ptosis, and Gellert-Holtzman score, compared to the controls. Statistically significant (*P* < 0.05 and *P* < 0.01) differences were also observed between morphine-treated groups in the number of jumps, ptosis, diarrhea, and Gellert-Holtzman score.

### Morphine Encapsulation in Liposomes Reduces CPA Duration but not CPA Expression

**Figure 7:**
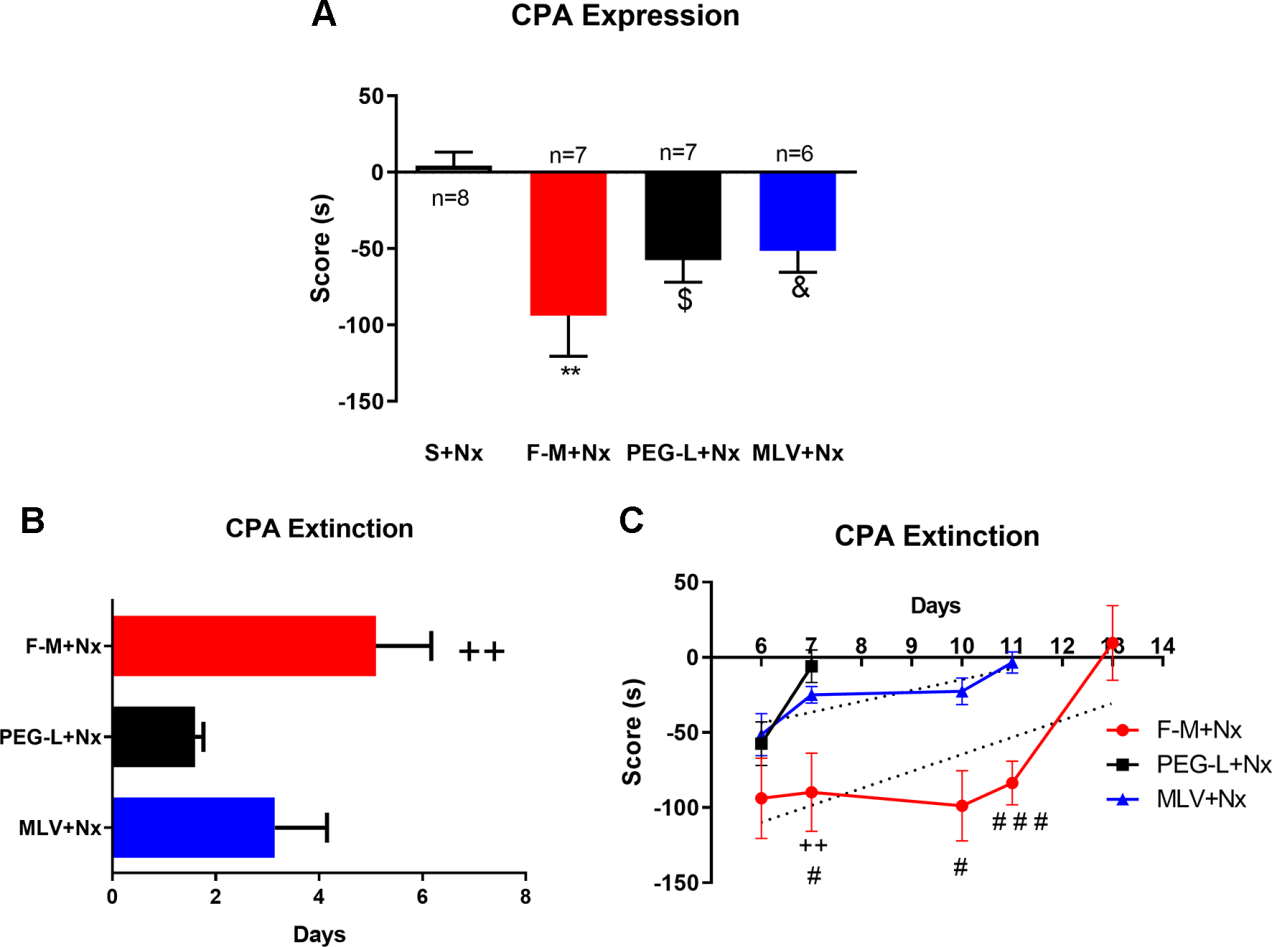
Morphine encapsulation in liposomes reduces conditioned-place aversion (CPA) duration but not CPA expression. **(A)** Effect of repeated saline or morphine injection on CPA test. Mice were treated with chronic morphine/saline for 4 days and received an acute injection of naloxone (1 mg/kg, s.c.). Morphine was administered encapsulated into MLV or PEG-L or in its free way (10–60 mg/kg; n = 8 per group) The score was calculated for each mouse as the difference between postconditioning and preconditioning time spent in the naloxone-paired compartment. **(B)** Maintenance of the CPA induced by a s.c. injection of naloxone to mice treated chronically with morphine, MLV, or PEG. The bars represent the number of days that each animal needed to achieve a complete extinction of the CPA (defined as a lack of significant differences with respect to preconditioning values). Data are the mean ± SEM.^ ++^ *P* < 0.01 versus PEG-L+Nx. **(C)** Extinction of CPA training. Aversion scores from day 6 to 13 are shown. Data are the mean ± SEM. **P < 0.01, ^$^ *P* < 0.05, ^&^ *P* < 0.05 versus S+Nx; ^++^ *P* < 0.01 versus PEG-L+Nx; ^#^ *P* < 0.05, ^###^ *P* < 0.01 versus MLV+Nx. Saline (S), naloxone (Nx), free morphine (F-M).

Morphine administration, either in its free form or encapsulated in MLV or PEG-L, induced a significant [*F*(3,24) = 6.078; *P* < 0.05, *P *< 0.01] degree of place aversion to the naloxone-paired chamber in comparison with the saline group (**Figure 7A**). Besides, one-way ANOVA for the CPA score indicated no differences between free morphine, MLV, and PEG-L.

To evaluate the persistence of CPA in free morphine–, PEG-L– and MLV-treated animals, this test was assessed every day until no significant differences in the time spent in the naloxone-paired room during two consecutive days, comparing to the pretest day, were observed. Animals receiving free morphine presented aversion for the naloxone-paired compartment for a longer time (5.10 days), followed by MLV (3.14 days) and PEG-L (1.60 days) (**Figure 7B**).

One-way ANOVA showed a significant increase in extinction days [*F*(2,24) = 4.979; *P* < 0.05] with statistically differences of free morphine comparing to PEG-L (*P* < 0.01) and with no differences between MLV and PEG-L (**Figure 7B**). These results also indicate that the time necessary to extinguish CPA was 1.5-fold higher in the free-morphine group, compared to MLV, and three folds higher than PEG-L. When comparing both liposome formulations, MLV produced a longer aversion (two folds) than PEG-L. Two-way ANOVA showed a main effect of kind of formulation [*F*(2,85) = 20.30, *P* < 0.0001] and day [*F*(4,85) = 7.843, *P* < 0.0001] with a significant interaction between kind of formulation and day [*F*(8,85) = 2.348, *P* = 0.0248] (**Figure 7C**). Moreover, *post hoc* analysis showed a significant increase in the aversion retained, on day 11 of free morphine–treated animals compared to MLV-treated animals (*P* < 0.001) (**Figure 7C**).

## Discussion

Morphine is an important pain reliever, which is widely used in cases of severe and chronic pain associated with heart attacks, serious injury, postoperative discomfort, and terminal illness such as cancer (Sverrisdottir et al., 2015). However, it is necessary to optimize the pharmaceutical forms used to supply patients with this drug, so that an effective dose could be reached while extending the intervals between its administrations. When selecting a suitable controlled drug delivery system for a particular drug, many factors including the physicochemical properties of the drug can influence, such as duration of release and the release model (Maherani et al., 2011).

In this study free morphine was compared with MLV and PEG-L, and in both liposomes formulations, we used remote loading to obtain a high level of drug entrapment. Cholesterol was included in both formulations to increase their stability (Hernandez-Caselles et al., 1990).

The pharmacokinetics study shows that morphine encapsulation, either in PEG-L or MLV, results in the controlled release of the encapsulated material. These results are consistent with other studies using epidural morphine administration in liposomes (Yaksh et al., 1999). Indeed, PEG-L AUC was 1.33-fold higher than MLV one, showing a little higher systemic bioavailability compared to MLV, but even higher (7.28-fold) if PEG-L is compared to free morphine at a dose of 90 mg/kg. After morphine has been released from the liposome and is absorbed systemically, morphine metabolism is thought to be the same as for other morphine formulations. So, it is estimated that the formation of morphine metabolites (M3G, M6G), would be similar to an i.p. injection of nonencapsulated morphine (De Gregori et al., 2012).

In our study, liposomes formulations ensured an extended-release drug delivery and morphine still remained in circulating blood 24 h after injection. Although only morphine was analyzed, morphine metabolites were expected to be detected during a longer time when using liposomes.

The differences between PEG-L and MLV pharmacokinetics could be explained by their different sizes and zeta potentials. Multilamellar vesicle is much larger than PEG-L, and thus, they must remain more time in the peritoneal zone. Meanwhile, PEG-L show a better zeta potential and greater stability, avoiding the reticular system and also disappearing from the circulation more slowly than MLV.

*In vitro* assays at 37°C showed a slower morphine release rate for PEG-L compared with MLV, during the first days of storage, and 50% of the morphine was released after 12 h in the case of MLV but after 24 h in the case of PEG-L. However, at day 7, the cumulative release of both preparations was the same (80%). Much more pronounced differences appeared when the experiments were carried out at 4°C, since the initial drug release was notably faster in the case of MLV. Furthermore, total drug release, after 60 days, was about 60% in the case of MLV compared to only 30% for PEG-L, indicating the lower stability of MLV at this temperature.

Different nociceptive tests can be used to measure pain sensitivity, chemical stimuli (visceral pain) and thermal stimuli (somatic pain), and it is thought that different mechanisms are involved in these tests. Whereas chemical stimuli are often used for the study of peripheral antinociception, and pain is indirectly produced through endogenous mediators, such as bradykinin, serotonin, histamine, substance P, PGs, and cytokines (Le Bars et al., 2001), the hot plate test, as well as tail flick, for example, is used to measure central pain, and spinal mechanisms are thought to be involved (Carsten, 2009). We used the hot plate test because we wanted to study the possible relationship between central pain and addiction. The difficulty to separate analgesia from addiction is preventing the resolution of the present question about using or not opioids to alleviate chronic pain. In some manner, pain relief requires the decoupling between powerful analgesia and addictive potential (Fields, 2011). Since empty liposomes and saline were compared in a preliminary test, and no differences were observed between them (data not shown), saline was chosen as a control for the antinociception study.

Multilamellar vesicle have the advantage of being very easily and quickly prepared, and in our hands, they show a very high entrapment capacity, but they are heterogeneous in size. On the other hand, PEG-L, in addition to be uniform in size, have as their main advantage that they induce a better morphine bioavailability, as mentioned above, and therefore better antinociceptive results. Other authors have used different types of morphine liposomes, such as sonicated liposomes, obtaining a longer antinociceptive effect: being 2.5 h for free morphine and extra 2 h when morphine was encapsulated in liposomes (Planas et al., 2000). In our case, a considerable prolongation of antinociception was obtained with MLV and PEG-L, compared with free morphine, which is related to the longer circulation time of morphine in blood obtained with both liposome formulations. In another work, freeze-thawed liposomes were used to encapsulate very high doses of morphine, providing analgesic effects of more than 20 h (Grant et al., 1994). Previous studies on antinociception (Grant et al., 1994) used liposomes to encapsulate high doses of morphine (doses interval 130–1650 mg/kg), but we decided to employ much more reduced doses trying to reflect what would be expected in a pharmacological use. Besides, the doses of 60 and 90 mg/kg are in the range of doses used in the behavioral studies. Thus, the conditioned-place aversion protocol uses escalating doses of morphine (10–60 mg/kg), a morphine protocol that has been widely used to study opioid tolerance and dependence (Garcia-Carmona et al., 2015).

In this experimental work, animal’s body weight during morphine treatment was measured, since several studies have shown that chronic morphine administration leads to a reduction in body weight gain because of a lower caloric intake (Garcia-Carmona et al., 2015; Martinez-Laorden et al., 2015). The present data agree with these results and confirm that morphine was correctly administered. Besides, no statistically significant differences were observed between free morphine and liposomal morphine administration.

Based on addiction research, the following question appears : What determines the progress from occasional drug use to abuse and addiction? Nowadays, it is well accepted that this change involves neuroplasticity and starts with a first drug use in predisposed individuals or in individuals at more vulnerable developmental stages (e.g., adolescence) (Koob and Le Moal, 2008). In the last few years, several publications have suggested that this vulnerability leads to persistent alterations in the behavior of the individual, which are due to significant changes in the gene expression (Kreek et al., 2012; Nestler, 2012). However, special features of drugs themselves could also promote the development of addiction.

In this study, differences in the rewarding properties of free morphine or morphine encapsulated in MLV or PEG-L were studied by means of CPP, an important behavioral test that is highly used to evaluate the rewarding properties of drugs and natural reinforcements. Our data show that morphine, administered in its free or encapsulated form, induces a significant CPP for the drug-associated compartment. These findings confirmed previous studies (Bali et al., 2015; Mohammadi et al., 2016) demonstrating that free morphine administration produced rewarding effects through an associative learning mechanism linked to the environment in which these effects were experienced. In contrast to our results using liposomal morphine, other authors demonstrated that the reinforcing properties of cocaine are modified according to the intravenous infusion speed (Samaha and Robinson, 2005). However, when the infusion rate of free opioids was modified, the reinforcing properties were not altered in agreement with our results obtained with morphine encapsulated in liposomes (Abreu et al., 2001). When comparing the resistance to CPP extinction between free morphine administration and morphine administered in the form of MLV or PEG-L, substantial differences were observed between groups in CPP extinction score: 43 days in the free-morphine group, 23 days for MLV and only 8 days in the case of PEG-L.

On the other hand, naloxone-precipitated withdrawal is often used in opiate addiction research because changes induced by this antagonist in morphine-dependent animals are more robust that changes induced by morphine deprivation (that would better reflect what happens in human addicts). Moreover, our results demonstrate that morphine administration either in a free or encapsulated way induces a robust CPA for the naloxone-paired place. These results agree with previous findings from our group using free morphine (Samaha and Robinson, 2005) and suggest that morphine withdrawal associated with negative affective consequences and place aversion to previous neutral environmental contexts could be involved in the maintenance of drug abuse (Budzynska et al., 2012). There is much information concerning the neurobiological mechanisms of the extinction of reward memory of drug consumption (Feltenstein and See, 2007; Hsu and Packard, 2008; Torregrossa et al., 2010), but less is known about the extinction of the negative memory of drug withdrawal (Myers and Carlezon, 2010). Although some authors have hypothesized that the fast drug access to the brain could induce addiction not simply because this makes drugs more euphorigenic or reinforcing, but because it facilitates their ability to induce neurobehavioral plasticity changes that contribute to compulsion in drug taking (Samaha and Robinson, 2005), there are no experimental data about morphine encapsulation and extinction memories. We found that the duration of CPA extinction score versus time is substantially reduced when animals are treated with morphine encapsulated in liposomes. Thus, CPA expression was evident in the free-morphine group until day 13, whereas the MLV group extinguished its aversion on day 11 and PEG-L on day 7.

Somatic signs of opiate withdrawal were recorded, and our results clearly reveal an increase in these signs in the free-morphine group injected with naloxone, similarly to what has been observed in other studies (Garcia-Carmona et al., 2015; García-Pérez et al., 2016). The MLV group showed a similar increase compared with the controls, although PEG-L–treated animals showed fewer signs of physical dependence.

Memory consolidation relates to the time-dependent stabilizing mechanism that brings to the long-term deposit of a new memory (McGaugh, 2000). Memory impairment during drug withdrawal is a complex process that demands the knowledge of the mechanisms that mediate the extinction of aversive memories, which could lead to therapeutic approaches for increasing extinction, facilitating drug addiction treatment (Garcia-Carmona et al., 2015). Overall, our data show that animals treated with encapsulated morphine present a reduced level of morphine-induced place preference and aversion, which could be due to an activation of the memory consolidation process in animals receiving free morphine. In our study, PEG-L and MLV enter the brain more slowly than free morphine, which may account for the differences in CPP and CPA extinction results. Moreover, our results point to a slower morphine release rate for PEG-L, which is correlated with the reduced persistence of positive or negative associations with drug-paired stimuli observed in this group. Our findings clearly support a similar role for PEG-L treatment in affective-like and somatic components of opioid withdrawal control. However, it is believed that somatic and affective signs of the opioid-withdrawal syndrome can be dissociated at different levels (Frenois et al., 2002), although the study in question only used morphine administered in its free form.

Our findings represent the first demonstration that morphine encapsulation in liposomes is associated with the process of memory consolidation and confirm that CPP and CPA share a common brain mechanism as it has been stated (Rosen et al., 2017). It can be suggested that the differences in the extinction times and, possibly, in specific molecular pathways are due to the speed of morphine entering into the brain. Nevertheless, it is premature to suggest a specific mechanism at this stage since a lot of research is still necessary to assess it. As shown here, animals receiving free morphine have an increased memory consolidation process that is reflected by a higher duration of CPP and CPA. Probably, the neurobehavioral changes induced by this drug would depend on the speed of delivery to the brain, as said before.

Recent results from our laboratory (not published), using the pattern of morphine administration used in our CPA study, have shown very interesting differences in several signaling pathways when comparing morphine liposomal formulations with free morphine. Therefore, we consider that a differential tolerance development, which will depend on the way in which morphine is administered, could be influencing our results (Garcia-Carmona et al., 2015).

Future studies are needed to identify the specific molecular pathways involved in these behavioral mechanisms.

Some other morphine encapsulated in liposomes has been described, and some of them are being used. For example, DepoDur is a preparation in which morphine sulfate, USP is encapsulated in multilamellar vesicles (Hartrick and Hartrick, 2008). The median diameter of the liposome particles is in the range of 17 to 23 µm, therefore bigger than the PEG-L ones used here. This form is designed for epidural administration, and it has not been reported to have been tested through other ways of administration. Therefore, it is difficult to compare their properties and effects with our preparations. To the best of our knowledge, the effect of this form of morphine on the facilitation of reward and aversive memories extinction has not been reported. The study of the differences in memories extinction shown in this work is a novelty, and in addition, our study shows the use of very well-characterized liposomes, as it is the case of PEG-L, which constitutes a very robust system, with high reproducibility and very extended life (slow drug release).

In summary, the results of this article point to an important advantage of using morphine encapsulated in liposomes, since this technological process could transform morphine into a potentially less addictive drug. In addition, our preclinical experiments performed in mice suggest that administering encapsulated morphine in liposomes could diminish the ability of this drug to induce dependence, as reflected by memory consolidation results. Our experiments represent a very new and innovative line of research, and we have to continue investigating this aspect in order to support strong conclusions. Based on our results, we could conclude that the encapsulation process provides a considerable long analgesic effect (as it has previously been described) and that morphine liposomes could attenuate the development of addiction and constitute a good alternative to the therapeutic use of this drug (Ali et al., 2017).

## Data Availability

Data used in this work are available upon request.

## Ethics Statement

All animal experiments were carried out in accordance with the European Communities Council Directive of 24 September 2010 (2010/63/UE) and were approved by the local committees for animal research (Comité Ético de Experimentación Animal, CEEA; Universidad de Murcia; RD 53/2013). Protocols were designed to minimize the number of experimental animals and to minimize their suffering. All methods were carried out in accordance with the relevant guidelines and regulations.

## Author Contributions

PA, BC, and JG-F conceived and designed the experiments; VG-M and BC performed the experiments and analyzed the data. PA, VG-M, and JG-F wrote the manuscript; ML and MM contributed to the design of some experiments.

## Funding

This research was supported by grants from the Ministerio de Ciencia e Innovación, Spain (SAF 2017-85679-R) and Fundación Séneca (20847/PI718), Comunidad Autónoma de la Región de Murcia (Spain).

## Conflict of Interest Statement

The authors declare that the research was conducted in the absence of any commercial or financial relationships that could be construed as a potential conflict of interest.
