# Supplementary material for: Liposome-Encapsulated Morphine Affords a Prolonged Analgesia While Facilitating Extinction of Reward and Aversive Memories
Source: Front Pharmacol. 2019 Sep 20;10:1082. doi: 10.3389/fphar.2019.01082 (PMC6764324; doi:10.3389/fphar.2019.01082)
Supplement: Supplementary file 1 [file DataSheet_1.pdf]

Supplementary Material

Liposome-encapsulated morphine affords a prolonged analgesia while facilitating extinction of reward and aversive memories.

Victoria Gómez-Murcia[1], Bruno Ribeiro Do Couto[2], Juan C. Gómez-Fernández[3*], María V. Milanés[1], María L. Laorden[1], Pilar Almela[1]

[1]Department of Pharmacology, Faculty of Medicine, University of Murcia, IMIB-Arrixaca, Spain. [2]Department of Human Anatomy and Psychobiology, Faculty of Psychology, University of Murcia, IMIB-Arrixaca, Spain. [3]Department of Biochemistry and Molecular Biology A, Faculty of Veterinary, Regional Campus of International Excellence "Campus Mare Nostrum", University of Murcia, IMIB-Arrixaca, Spain.

*Correspondence and requests for materials should be addressed to J.C.G-F (email: jcgomez@um.es).

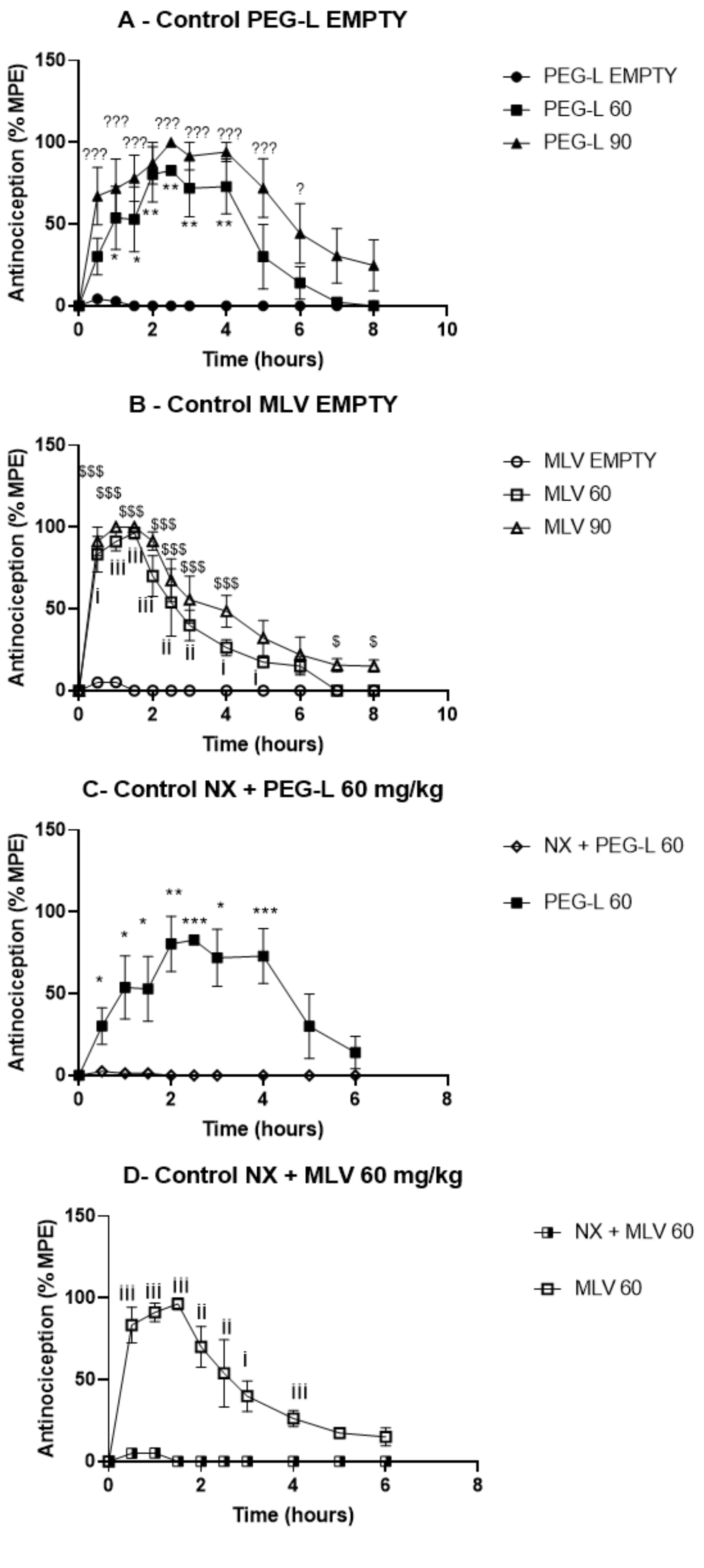

Figure supplementary 1. Morphine encapsulated in liposomes formulations are the responsible of the antinociception effect compared to controls. (**a**) Antinociceptive effect as a function of time after a single i.p. administration of PEG-L at 60 and 90 mg/kg compared to empty PEG-L. (**b**) Antinociceptive effect as a function of time after a single i.p. administration of MLV at 60 and 90 mg/kg compared to empty MLV. **(c)**

Antinociceptive effect as a function of time after a single i.p. administration of PEG-L at 60 mg/kg compared to NX + PEG-L 60 mg/kg. **(d)** Antinociceptive effect as a function of time after a single i.p. administration of MLV at 60 mg/kg compared to NX + MLV 60 mg/kg. The intensity of analgesia is expressed as % MPE (maximal potent effect). Each data point represents the average for 5-8 animals. Data are expressed as mean ± SEM. $^{?}P<0.05$, $^{???}P<0.001$; $^{*}P<0.05$, $^{**}P<0.01$, $^{***}P<0.001$ vs empty PEG-L and NX+ PEG-L. $^{\$}P<0.05$, $^{\$\$\$}P<0.001$; $^{i}P<0.05$, $^{ii}P<0.01$, $^{iii}P<0.001$ vs MLV empty and NX + MLV 60.
